# Cancer Incidence and Trends in Persistent Poverty Areas of California by Race/Ethnicity and Sex

**DOI:** 10.1002/cam4.71088

**Published:** 2025-07-30

**Authors:** Ani S. Movsisyan Vernon, Frances B. Maguire, Ayman T. Ullah, Brenda M. Hofer, Arti Parikh‐Patel, Ted Wun, Shehnaz K. Hussain, Theresa H. M. Keegan

**Affiliations:** ^1^ UC Davis Health University of California Davis Comprehensive Cancer Center Sacramento California USA

**Keywords:** California, cancer, incidence, persistent poverty, poverty, race, race/ethnicity

## Abstract

**Background:**

Although previous studies have observed the relationship between living in persistent poverty areas (PPAs) and adverse cancer outcomes, the relationship between residing in PPAs and disparities in cancer incidence rates and temporal trends by race/ethnicity and sex in California is unknown.

**Methods:**

We used California Cancer Registry data to identify patients diagnosed with 16 common cancers between 2006 and 2020. We calculated age‐adjusted incidence rates (IRs), incidence rate ratios (IRRs), and average annual percent changes (AAPCs) to compare incidence and temporal trends in PPAs and non‐PPAs in California by race/ethnicity and sex. PPA was defined as areas with a poverty rate of 20% or more for approximately 30 continuous years.

**Results:**

Male patients in PPAs had significantly higher IRRs of colorectal, liver, lung, and stomach cancers, and female patients had higher cervical, kidney, liver, and stomach cancers. Across all racial/ethnic groups, cervical and liver cancer IRRs were significantly higher, and IRRs for female breast cancer were lower in PPAs. The incidence of several cancers significantly decreased over time only in non‐PPAs. Colorectal cancer and non‐Hodgkin lymphoma among Hispanic residents significantly increased in PPAs (AAPC = 0.4, 1.2) and colorectal cancer decreased only in non‐PPAs (AAPC = −1.4). Thyroid cancer among non‐Hispanic Black residents increased only in PPAs (AAPC = 4.7).

**Conclusion:**

Our findings highlight race/ethnicity and sex disparities in cancer incidence and temporal trends for PPA residents. Further research is needed to understand the specific etiological factors underlying these disparities, and to support the development of public health policies and initiatives to decrease the cancer burden in California.

## 1 Introduction

Disparities in cancer incidence continue in California regions with a high prevalence of low socioeconomic indicators and racial/ethnic minority populations [1, 2]. Persistent poverty areas (PPAs), defined as regions with a poverty rate of 20% or more for approximately 30 continuous years [3], have larger proportions of historically underrepresented racial/ethnic residents, higher concentrations of environmental, occupational, and infectious carcinogens, higher rates of food and housing insecurity, and less access to transportation compared to non‐PPAs [4, 5, 6].

Long‐term deprivation of resources and opportunities experienced by people in poverty is linked with worse cancer outcomes due to factors including barriers to healthcare access, delayed diagnosis, and greater exposure to environmental toxins [7]. Although populations in PPAs have higher mortality from multiple cancer types, including lung, colorectal, stomach, and liver cancers, incidence patterns for common cancers in PPAs by race/ethnicity and sex are not yet well established in literature [8, 9]. As such, the National Cancer Institute (NCI) has recently launched initiatives advocating for efforts to study and address observed cancer disparities in PPAs across the United States [4, 7, 10].

According to the 2010 Census population estimates, higher percentages of Hispanic, non‐Hispanic Black, American Indian/Alaska Native, foreign‐born, and younger residents reside in California PPAs than non‐PPAs [11]. As of 2023, nearly a third of California residents live in or near poverty in California, with higher poverty rates observed among Hispanic individuals, adults with low educational attainment, and foreign‐born individuals [2, 5, 12]. However, California's social safety net programs, such as CalFresh, CalWORKs, and the Federal Child Tax Credit, have led to overall reduced poverty improvements, preventing an estimated three million more Californians from living in poverty [12].

Understanding cancer incidence patterns by race/ethnicity and sex in the context of poverty in California, the largest and most culturally diverse state in the country, is important because social, economic, and political discriminatory systems in the United States have historically marginalized specific populations based on characteristics such as race/ethnicity and sex [7, 13, 14]. Although several studies have examined cancer incidence patterns and highlighted barriers to cancer prevention in PPAs [6, 8, 15, 16, 17, 18], this study extends these findings by observing cancer incidence by race/ethnicity and sex in California. Specifically, we identified patients diagnosed with 16 common cancers between 2006 and 2020 and compared incidence rates and temporal trends in PPAs and non‐PPAs in California by race/ethnicity and sex. Understanding cancer incidence and trends in PPAs can guide healthcare providers, public health researchers, and policymakers toward evidence‐based initiatives to prevent cancer among Californians living in impoverished areas of the state.

## 2 Methods

### 2.1 Data Sources

The California Cancer Registry (CCR) is a statewide population‐based cancer surveillance system and has collected cancer diagnoses in California since 1988 [19, 20]. The CCR is Gold Certified by the North American Association of Central Cancer Registries (NAACCR), meets the highest data quality standards, and is one of the largest cancer registries in the world [19, 21]. The CCR provides patient demographics and tumor characteristics including primary site and histology. For the present analyses, we considered year of diagnosis, age at diagnosis, sex, race/ethnicity, tumor primary site, and tumor histology. This study includes 16 common malignant cancers diagnosed in California between January 1, 2006 and December 31, 2020. Cancers were classified based on site and histology using the NCI's Surveillance, Epidemiology and End Results (SEER) site recode [22].

### 2.2 Persistent Poverty Measure and California Counties and Census Tracts

The United States Department of Agriculture (USDA) Economic Research Service (ERS) is the primary source of information used to derive persistent poverty area measures used for research including by the NCI [23]. The USDA ERS defines PPAs as areas in which 20% or greater of the population have lived in poverty for four consecutive time periods, 10 years apart, and persisting for approximately 30 years [3]. The USDA ERS provides PPA data for counties and census tracts across the United States that we accessed using the University of California San Francisco (UCSF) Health Atlas project, a resource provided by the UCSF Office of Population Health and Health Equity that provides persistent poverty status of census tracts in California based on 2010 Census boundaries [3, 11]. Based on the patient's census tract of residence at diagnosis, we used UCSF Health Atlas data to assign PPA status (PPA, non‐PPA) to each cancer, and to append PPA status to census tract‐level population estimates. There were 8057 census tracts in the state as of the 2010 Census [24, 25]. We obtained annual census tract population estimates by age group, sex, and race/ethnicity for the years 2006 through 2020 from the NCI [24]. We used census tracts as the geographic unit of analysis in this study.

### 2.3 Statistical Analyses

Using SEER*Stat (version 8.4.4) [26], we calculated age‐adjusted incidence rates (IRs) per 100,000 people adjusted to the 2000 U.S. standard population. To compare IRs by race/ethnicity and sex categories in PPAs with non‐PPAs, we calculated incidence rate ratios (IRRs), respective 95% confidence intervals, and *p* values, with non‐PPAs as the reference group. Analyses by sex were based on the availability of population denominators among male and female sex categories only. For female breast, cervical, and uterine cancer analyses, we used the female population at risk, and for prostate cancer analyses, we used the male population at risk. Breast cancer rates for males were included in analyses stratified by sex. Analyses by race/ethnicity included American Indian, Asian/Pacific Islander, non‐Hispanic Black, Hispanic, and non‐Hispanic White groups. To evaluate temporal trends of incidence rates, we used Joinpoint software (version 4.9.1) [27] to calculate the average annual percent change (AAPC), which is a single summary measure of the trend over a pre‐specified fixed time interval of multiple years. The AAPC represents the weighted average of annual percent changes in cancer incidence rates per year over the specified time interval, allowing for a direct comparison of incidence trends between our study population subgroups [28, 29]. Due to restricted healthcare access related to the COVID‐19 pandemic, observed cancer incidence was lower than expected in 2020 [30]; therefore, we calculated incidence trends for the period between 2006 and 2019. Trends for some subgroups could not be calculated in PPAs due to small populations and unstable underlying incidence rates.

## 3 Results

### 3.1 Patient Characteristics

During 2006–2020, 2,493,936 individuals were diagnosed with malignant cancer and 162,538 (6.5%) lived in PPAs at the time of diagnosis. The largest proportion (41.3%) of patients in PPAs were Hispanic, whereas the largest proportion (63.1%) of patients in non‐PPAs were non‐Hispanic White (Table 1). Roughly one‐third of patients in PPAs lived in Central California (32.1%) or in the Los Angeles area (32.6%). Fewer patients in PPAs had private insurance (38.5% vs. 60.4%) and more had public insurance or were uninsured (54.9% vs. 33.4%) compared to those in non‐PPAs. There were notably higher proportions of several cancer types in PPAs compared to non‐PPAs including cervical (1.7% vs. 0.8%), colorectal (9.9% vs. 8.8%), kidney (4.0% vs. 3.5%), liver (3.9% vs. 2.3%), lung (11.2% vs. 10.2%), and stomach (2.7% vs. 1.7%) cancers. Cancers with notably higher proportions in non‐PPAs compared to PPAs included breast (15.7% vs. 13.2%), bladder (4.1% vs. 3.1%), melanoma (5.4% vs. 2.1%), and prostate (12.8% vs. 11.1%).

**TABLE 1:** Characteristics of cancer patients by persistent poverty area (PPA), California Cancer Registry, 2006–2020.

Patient characteristics	Total (2,493,936)	PPA (162,538)	Non‐PPA (2,331,398)
	*N* (%)	*n* (%)	*n* (%)
*Age (years)*			
0–14	19,201 (0.8)	2355 (1.4)	16,846 (0.7)
15–39	132,632 (5.3)	12,175 (7.5)	120,457 (5.2)
40–64	976,466 (39.2)	69,256 (42.6)	907,210 (38.9)
65–79	938,733 (37.6)	55,830 (34.3)	882,903 (37.9)
80 plus	426,904 (17.1)	22,922 (14.1)	403,982 (17.3)
*Sex*			
Female	1,242,142 (49.8)	79,957 (49.2)	1,162,185 (49.8)
Male	1,251,794 (50.2)	82,581 (50.8)	1,169,213 (50.2)
*Health insurance status*			
Private	1,469,681 (58.9)	62,569 (38.5)	1,407,112 (60.4)
Public/uninsured	867,748 (34.8)	89,165 (54.9)	778,583 (33.4)
Unknown	156,507 (6.3)	10,804 (6.6)	145,703 (6.2)
*Race/ethnicity*			
American Indian	14,703 (0.6)	1536 (0.9)	13,167 (0.6)
Asian/Pacific Islander	278,188 (11.2)	16,212 (10.0)	261,976 (11.2)
Hispanic	477,242 (19.1)	67,107 (41.3)	410,135 (17.6)
Non‐Hispanic Black	159,296 (6.4)	25,504 (15.7)	133,792 (5.7)
Non‐Hispanic White	1,521,974 (61.0)	50,347 (31.0)	1,471,627 (63.1)
Other/unknown	42,533 (1.7)	1832 (1.1)	40,701 (1.8)
*Area of California*			
Bay Area	484,609 (19.4)	13,150 (8.1)	471,459 (20.2)
Central California	574,232 (23.0)	52,191 (32.1)	522,041 (22.4)
Los Angeles	594,845 (23.9)	52,924 (32.6)	541,921 (23.2)
Northern California	408,085 (16.4)	26,060 (16.0)	382,025 (16.4)
San Diego, Imperial	432,165 (17.3)	18,213 (11.2)	413,952 (17.8)
*Cancer type*			
Bladder	101,414 (4.1)	5091 (3.1)	96,323 (4.1)
Breast	388,005 (15.6)	21,472 (13.2)	366,533 (15.7)
Cervical	22,112 (0.9)	2767 (1.7)	19,345 (0.8)
Colorectal	221,005 (8.9)	16,109 (9.9)	204,896 (8.8)
Kidney	86,993 (3.5)	6506 (4.0)	80,487 (3.5)
Leukemia	74,817 (3.0)	5305 (3.3)	69,512 (3.0)
Liver	59,520 (2.4)	6334 (3.9)	53,186 (2.3)
Lung	256,261 (10.3)	18,280 (11.2)	237,981 (10.2)
Melanoma	130,363 (5.2)	3392 (2.1)	126,971 (5.4)
Non‐Hodgkin lymphoma	109,611 (4.4)	7017 (4.3)	102,594 (4.4)
Oral	62,882 (2.5)	4060 (2.5)	58,822 (2.5)
Pancreas	70,415 (2.8)	4669 (2.9)	65,746 (2.8)
Prostate	316,062 (12.7)	18,093 (11.1)	297,969 (12.8)
Stomach	44,474 (1.8)	4420 (2.7)	40,054 (1.7)
Thyroid	71,509 (2.9)	4528 (2.8)	66,981 (2.9)
Uterine	82,241 (3.3)	5626 (3.5)	76,615 (3.3)
Other	396,252 (15.9)	28,869 (17.8)	367,383 (15.8)

Abbreviations: Non‐PPA: non‐persistent poverty area; PPA, persistent poverty area.

### 3.2 Cancer Incidence Rates, 2006–2020

In Table 2, we show cancer incidence rates among males and females in PPAs compared to non‐PPAs, with statistically significant differences highlighted below. The rate for all cancers combined was lower among males living in PPAs (IRR = 0.92, 95% CI = 0.91, 0.93, *p <* 0.001) compared to non‐PPAs. The same pattern was observed among females (IRR = 0.88, 95% CI = 0.88, 0.89, *p <* 0.001), although the cancer‐site specific associations differed by sex. Males in PPAs had higher rates of colorectal (IRR = 1.07, 95% CI = 1.04, 1.09, *p <* 0.001), liver (IRR = 1.54, 95% CI = 1.49, 1.59, *p <* 0.001), lung (IRR = 1.14, 95% CI = 1.12, 1.16, *p <* 0.001), and stomach (IRR = 1.43, 95% CI = 1.37, 1.49, *p <* 0.001) cancers compared to their counterparts in non‐PPAs. Females in PPAs had higher rates of cervical (IRR = 1.63, 95% CI = 1.56, 1.69, *p <* 0.001), kidney (IRR = 1.14, 95% CI = 1.09, 1.19, *p <* 0.001), liver (IRR = 1.57, 95% CI = 1.49, 1.65, *p <* 0.001), and stomach (IRR = 1.49, 95% CI = 1.42, 1.57, *p* < 0.001) cancers compared to those in non‐PPAs.

**TABLE 2:** Age‐adjusted incidence rates (IR) and rate ratios (IRR) by persistent poverty area (PPA) and sex, 2006–2020.

Sex/cancer type	PPA		Non‐PPA		PPA/non‐PPA		
	*N*	IR	*N*	IR	IRR	95% CI	*p*
*Male*							
All cancers	80,950	412.5	1,133,804	447.8	0.92*	(0.91, 0.93)	< 0.001
Bladder	3838	22.2	74,076	31.1	0.71*	(0.69, 0.74)	< 0.001
Breast	167	0.9	2586	1.0	0.84*	(0.71, 0.98)	0.03
Colorectal	8769	45.1	106,607	42.3	1.07*	(1.04, 1.09)	< 0.001
Kidney	3945	19.5	51,617	20.0	0.98	(0.94, 1.01)	0.15
Leukemia	3074	13.8	40,490	16.5	0.84*	(0.81, 0.88)	< 0.001
Liver	4505	21.6	37,486	14.0	1.54*	(1.49, 1.59)	< 0.001
Lung	10,175	56.3	119,821	49.4	1.14*	(1.12, 1.16)	< 0.001
Melanoma of the skin	1985	10.3	76,410	30.6	0.34*	(0.32, 0.35)	< 0.001
Non‐Hodgkin lymphoma	3857	19.2	56,921	22.8	0.84*	(0.81, 0.87)	< 0.001
Oropharyngeal	2902	14.0	41,291	15.7	0.89*	(0.86, 0.93)	< 0.001
Pancreas	2379	12.7	33,432	13.5	0.94*	(0.90, 0.98)	0.007
Prostate	18,092	95.0	297,969	113.1	0.84*	(0.83, 0.85)	< 0.001
Stomach	2579	13.7	23,820	9.6	1.43*	(1.37, 1.49)	< 0.001
Thyroid	1007	4.4	16,705	6.4	0.69*	(0.64, 0.74)	< 0.001
*Female*							
All cancers	79,517	346.4	1,151,970	391.9	0.88*	(0.88, 0.89)	< 0.001
Bladder	1253	5.7	22,247	7.3	0.78*	(0.74, 0.83)	< 0.001
Breast	21,305	93.4	363,947	124.7	0.75*	(0.74, 0.76)	< 0.001
Cervix	2767	11.7	19,345	7.2	1.63*	(1.56, 1.69)	< 0.001
Colorectal	7340	32.5	98,289	32.8	0.99	(0.97, 1.02)	0.54
Kidney	2561	11.2	28,870	9.8	1.14*	(1.09, 1.19)	< 0.001
Leukemia	2231	8.9	29,022	10.1	0.88*	(0.84, 0.92)	< 0.001
Liver	1829	8.1	15,700	5.2	1.57*	(1.49, 1.65)	< 0.001
Lung	8105	36.9	118,160	39.3	0.94*	(0.92, 0.96)	< 0.001
Melanoma of the skin	1407	6.0	50,561	17.5	0.34*	(0.32, 0.36)	< 0.001
Non‐Hodgkin lymphoma	3160	13.8	45,673	15.5	0.89*	(0.86, 0.93)	< 0.001
Oropharyngeal	1158	5.0	17,531	5.9	0.85*	(0.79, 0.90)	< 0.001
Pancreas	2290	10.3	32,314	10.6	0.97	(0.93, 1.02)	0.22
Stomach	1,841	8.2	16,234	5.5	1.49*	(1.42, 1.57)	< 0.001
Thyroid	3521	14.2	50,276	18.6	0.76*	(0.73, 0.79)	< 0.001
Uterine	5626	24.2	76,614	25.4	0.95*	(0.93, 0.98)	< 0.001

Abbreviations: CI, confidence interval; IR, age‐adjusted incidence rate per 100,000 people; IRR, incidence rate ratio (PPA/non‐PPA); non‐PPA, non‐persistent poverty area; PPA, persistent poverty area.

* The IRR is statistically significant at *p* < 0.05.

In Table 3, we show cancer incidence rates in PPAs compared to non‐PPAs by race/ethnicity with statistically significant results highlighted below. We observed higher incidence rates of lung cancer among non‐Hispanic Black and non‐Hispanic White residents compared to other racial/ethnic groups, with higher rates occurring among those living in PPAs. The results for all cancers show that non‐Hispanic Black (IRR = 1.09, 95% CI = 1.07, 1.10, *p <* 0.001) and non‐Hispanic White (IRR = 1.05, 95% CI = 1.04, 1.06, *p <* 0.001) residents in PPAs had higher incidence relative to residents of the same race/ethnicity in non‐PPAs. On the other hand, Asian/Pacific Islander (IRR = 0.94, 95% CI = 0.93, 0.96, *p <* 0.001) and Hispanic (IRR = 0.92, 95% CI = 0.91, 0.92, *p <* 0.001) residents living in PPAs had lower overall cancer incidence rates than the same race/ethnicity groups in non‐PPAs.

**TABLE 3:** Age‐adjusted incidence rates (IR) and rate ratios (IRR) by persistent poverty area (PPA) and race/ethnicity, 2006–2020.

Cancer type	PPA		Non‐PPA		IRR	95% CI	*p*
	*N*	IR	*N*	IR			
*American Indian*							
All cancers	1522	437.5	13,006	443.0	0.99	(0.93, 1.04)	0.67
Bladder	46	13.4	422	15.5	0.87	(0.62, 1.18)	0.41
Cervix	33	18.9	164	11.7	1.62*	(1.07, 2.38)	0.02
Colorectal	158	45.0	1192	41.7	1.08	(0.90, 1.27)	0.41
Female breast	201	102.3	2048	129.4	0.79*	(0.68, 0.92)	0.002
Kidney	101	28.9	649	21.3	1.36*	(1.08, 1.69)	0.009
Leukemia	46	13.9	417	14.8	0.94	(0.67, 1.29)	0.77
Liver	107	28.5	634	19.6	1.46*	(1.17, 1.80)	0.001
Lung	176	51.7	1373	49.0	1.06	(0.89, 1.24)	0.54
Melanoma	18	5.0	365	12.5	0.40*	(0.23, 0.65)	< 0.001
Non‐Hodgkin lymphoma	63	19.3	523	18.4	1.05	(0.79, 1.37)	0.77
Oropharyngeal	37	10.8	369	11.8	0.92	(0.63, 1.30)	0.69
Pancreas	34	9.6	380	13.4	0.72	(0.48, 1.03)	0.07
Prostate	118	78.9	1317	90.6	0.87	(0.71, 1.06)	0.18
Stomach	17	4.8	219	7.4	0.65	(0.36, 1.07)	0.1
Thyroid	35	10.1	368	12.9	0.79	(0.53, 1.12)	0.21
Uterine	65	32.4	537	33.3	0.97	(0.73, 1.27)	0.91
*Asian/Pacific Islander*							
All cancers	16,031	289.2	258,785	306.1	0.94*	(0.93, 0.96)	< 0.001
Bladder	470	7.8	6999	8.6	0.90*	(0.82, 0.99)	0.03
Cervix	295	11.2	3028	6.5	1.72*	(1.52, 1.95)	< 0.001
Colorectal	2130	37.6	28,315	33.4	1.12*	(1.07, 1.18)	< 0.001
Female breast	2239	82.1	50,130	104.9	0.78*	(0.75, 0.82)	< 0.001
Kidney	400	7.4	7131	8.3	0.88*	(0.80, 0.98)	0.02
Leukemia	388	7.0	6408	8.0	0.88*	(0.79, 0.97)	0.01
Liver	962	17.4	11,403	13.4	1.29*	(1.21, 1.38)	< 0.001
Lung	2235	38.3	29,072	35.3	1.09*	(1.04, 1.13)	< 0.001
Melanoma	51	0.9	1025	1.2	0.75*	(0.55, 1.00)	0.04
Non‐Hodgkin lymphoma	642	11.4	11,781	14.2	0.80*	(0.74, 0.87)	< 0.001
Oropharyngeal	436	8.3	6610	7.7	1.08	(0.98, 1.20)	0.12
Pancreas	540	9.3	8,104	9.9	0.94	(0.86, 1.02)	0.16
Prostate	1051	41.1	24,256	64.3	0.64*	(0.60, 0.68)	< 0.001
Stomach	766	13.2	8317	10.1	1.31*	(1.21, 1.41)	< 0.001
Thyroid	545	10.6	11,626	13.6	0.78*	(0.71, 0.85)	< 0.001
Uterine	535	19.3	10,602	21.6	0.90*	(0.82, 0.98)	0.02
*Non‐Hispanic Black*							
All cancers	25,275	481.9	132,413	442.8	1.09*	(1.07, 1.10)	< 0.001
Bladder	692	13.7	3556	12.9	1.06	(0.98, 1.16)	0.14
Cervix	292	10.9	1073	6.8	1.60*	(1.39, 1.82)	< 0.001
Colorectal	2669	51.6	13,173	45.2	1.14*	(1.09, 1.19)	< 0.001
Female breast	3426	122.0	21,031	129.5	0.94*	(0.91, 0.98)	0.001
Kidney	938	17.9	5432	17.9	1.00	(0.93, 1.07)	0.99
Leukemia	584	11.3	3168	11.0	1.03	(0.94, 1.12)	0.58
Liver	890	15.8	3310	10.4	1.53*	(1.42, 1.65)	< 0.001
Lung	3866	74.4	15,447	54.0	1.38*	(1.33, 1.43)	< 0.001
Melanoma	44	0.8	312	1.1	0.75	(0.53, 1.03)	0.08
Non‐Hodgkin lymphoma	756	14.5	4421	14.9	0.98	(0.90, 1.06)	0.59
Oropharyngeal	623	11.5	2591	8.4	1.38*	(1.26, 1.51)	< 0.001
Pancreas	861	16.6	4431	15.5	1.07	(1.00, 1.16)	0.07
Prostate	4021	165.8	25,504	178.7	0.93*	(0.90, 0.96)	< 0.001
Stomach	596	11.7	2581	9.2	1.27*	(1.16, 1.39)	< 0.001
Thyroid	331	6.4	2304	7.5	0.85*	(0.76, 0.96)	0.007
Uterine	785	26.7	4486	26.4	1.01	(0.94, 1.09)	0.76
*Hispanic*							
All cancers	66,559	308.4	405,506	336.6	0.92*	(0.91, 0.92)	< 0.001
Bladder	1408	8.2	10,255	10.2	0.80*	(0.75, 0.85)	< 0.001
Cervix	1652	12.2	6768	9.0	1.36*	(1.28, 1.44)	< 0.001
Colorectal	6450	31.3	38,966	33.4	0.94*	(0.91, 0.96)	< 0.001
Female breast	9152	75.7	64,920	94.3	0.80*	(0.79, 0.82)	< 0.001
Kidney	3,359	15.5	20,583	16.8	0.92*	(0.89, 0.96)	< 0.001
Leukemia	2832	9.8	14,520	10.4	0.94*	(0.89, 0.98)	0.005
Liver	3066	15.0	15,176	13.1	1.14*	(1.09, 1.19)	< 0.001
Lung	4154	24.0	25,261	25.2	0.95*	(0.92, 0.99)	0.006
Melanoma	615	2.8	6201	5.0	0.57*	(0.52, 0.62)	< 0.001
Non‐Hodgkin lymphoma	3,470	16.4	20,720	17.5	0.94*	(0.90, 0.97)	< 0.001
Oropharyngeal	1232	5.7	7547	6.2	0.91*	(0.85, 0.97)	0.005
Pancreas	1839	9.9	11,817	11.2	0.89*	(0.84, 0.94)	< 0.001
Prostate	6983	82.7	47,635	97.3	0.85*	(0.83, 0.87)	< 0.001
Stomach	2331	11.5	11,424	10.1	1.14*	(1.09, 1.20)	< 0.001
Thyroid	2636	9.3	17,945	11.8	0.79*	(0.75, 0.82)	< 0.001
Uterine	2724	22.0	15,957	22.8	0.96	(0.92, 1.01)	0.09
*Non‐Hispanic White*							
All cancers	49,287	478.6	1,436,402	456.5	1.05*	(1.04, 1.06)	< 0.001
Bladder	2424	22.8	73,582	22.2	1.03	(0.99, 1.07)	0.17
Cervix	476	11.9	8097	6.6	1.81*	(1.64, 2.00)	< 0.001
Colorectal	4595	44.3	120,982	38.0	1.17*	(1.13, 1.20)	< 0.001
Female breast	6115	121.5	222,390	140.2	0.87*	(0.84, 0.89)	< 0.001
Kidney	1679	16.7	46,167	14.7	1.13*	(1.08, 1.19)	< 0.001
Leukemia	1408	14.2	43,953	14.6	0.98	(0.92, 1.03)	0.38
Liver	1293	12.0	22,484	6.7	1.77*	(1.68, 1.88)	< 0.001
Lung	7787	73.5	166,177	50.3	1.46*	(1.43, 1.50)	< 0.001
Melanoma	2458	24.6	109,604	36.3	0.68*	(0.65, 0.71)	< 0.001
Non‐Hodgkin lymphoma	1994	19.7	63,526	20.4	0.97	(0.92, 1.01)	0.16
Oropharyngeal	1686	16.4	40,909	12.8	1.28*	(1.21, 1.34)	< 0.001
Pancreas	1381	13.1	40,846	12.3	1.06*	(1.00, 1.12)	0.04
Prostate	5295	103.0	186,288	116.9	0.88*	(0.86, 0.91)	< 0.001
Stomach	698	6.7	17,281	5.3	1.26*	(1.17, 1.36)	< 0.001
Thyroid	918	9.9	33,896	13.2	0.75*	(0.70, 0.80)	< 0.001
Uterine	1468	28.4	44,342	26.2	1.09*	(1.03, 1.15)	0.003

Abbreviations: CI, confidence interval; IR, age‐adjusted incidence rate per 100,000 people; IRR, incidence rate ratio (PPA/non‐PPA); non‐PPA, non‐persistent poverty area; PPA, persistent poverty area.

* The IRR is statistically significant at *p* < 0.05.

Specific to American Indian residents, notable results include higher rates of cervical (IRR = 1.62, 95% CI = 1.07, 2.38, *p* = 0.02), kidney (IRR = 1.36, 95% CI = 1.08, 1.69, *p* = 0.009), and liver (IRR = 1.46, 95% CI = 1.17, 1.80, *p* = 0.001) cancers and lower rates of female breast cancer (IRR = 0.79, 95% CI = 0.68, 0.92, *p* = 0.002) and melanoma (IRR = 0.40, 95% CI = 0.23, 0.65, *p <* 0.001) among those in PPAs compared to those in non‐PPAs (Table 3).

Asian/Pacific Islander residents in PPAs had higher rates of cervical (IRR = 1.72, 95% CI = 1.52, 1.95, *p <* 0.001), colorectal (IRR = 1.12, 95% CI = 1.07, 1.18, *p <* 0.001), liver (IRR = 1.29, 95% CI = 1.21, 1.38, *p <* 0.001), lung (IRR = 1.09, 95% CI = 1.04, 1.13, *p <* 0.001), and stomach (IRR = 1.31, 95% CI = 1.21, 1.41, *p <* 0.001) cancers (Table 3). However, residents in PPAs had lower rates for most cancers we studied including bladder (IRR = 0.90, 95% CI = 0.82, 0.99, *p* = 0.03), female breast (IRR = 0.78, 95% CI = 0.75, 0.82, *p <* 0.001), kidney (IRR = 0.88, 95% CI = 0.80, 0.98, *p* = 0.02), leukemia (IRR = 0.88, 95% CI = 0.79, 0.97, *p* = 0.01), melanoma (IRR = 0.75, 95% CI = 0.55, 1.00, *p* = 0.04), non‐Hodgkin lymphoma (IRR = 0.80, 95% CI = 0.74, 0.87, *p <* 0.001), prostate (IRR = 0.64, 95% CI = 0.60, 0.68, *p <* 0.001), thyroid (IRR = 0.78, 95% CI = 0.71, 0.85, *p <* 0.001), and uterine (IRR = 0.90, 95% CI = 0.82, 0.98, *p* = 0.02) cancers.

Among non‐Hispanic Black residents, we observed higher rates of cervical (IRR = 1.60, 95% CI = 1.39, 1.82, *p <* 0.001), colorectal (IRR = 1.14, 95% CI = 1.09, 1.19, *p <* 0.001), liver (IRR = 1.53, 95% CI = 1.42, 1.65, *p <* 0.001), lung (IRR = 1.38, 95% CI = 1.33, 1.43, *p <* 0.001), oropharyngeal (IRR = 1.38, 95% CI = 1.26, 1.51, *p <* 0.001), and stomach (IRR = 1.27, 95% CI = 1.16, 1.39, *p <* 0.001) cancers and lower rates of female breast (IRR = 0.94, 95% CI = 0.91, 0.98, *p* = 0.001), prostate (IRR = 0.93, 95% CI = 0.90, 0.96, *p <* 0.001), and thyroid (IRR = 0.85, 95% CI = 0.76, 0.96, *p* = 0.007) cancers (Table 3).

For the Hispanic group, our results show lower rates for most cancers in PPAs versus non‐PPAs including bladder (IRR = 0.80, 95% CI = 0.75, 0.85, *p* < 0.001), colorectal (IRR = 0.94, 95% CI = 0.91, 0.96, *p* < 0.001), female breast (IRR = 0.80, 95% CI = 0.79, 0.82, *p <* 0.001), kidney (IRR = 0.92, 95% CI = 0.89, 0.96, *p <* 0.001), leukemia (IRR = 0.94, 95% CI = 0.89, 0.98, *p* = 0.005), lung (IRR = 0.95, 95% CI = 0.92, 0.99, *p* = 0.006), melanoma (IRR = 0.57, 95% CI = 0.52, 0.62, *p* < 0.001), non‐Hodgkin lymphoma (IRR = 0.94, 95% CI = 0.90, 0.97, *p* < 0.001), oropharyngeal (IRR = 0.91, 95% CI = 0.85, 0.97, *p* = 0.005), pancreatic (IRR = 0.89, 95% CI = 0.84, 0.94, *p* < 0.001), prostate (IRR = 0.85, 95% CI = 0.83, 0.87, *p* < 0.001), and thyroid (IRR = 0.79, 95% CI = 0.75, 0.82, *p* < 0.001) cancers (Table 3). However, our findings also showed higher rates of cervical (IRR = 1.36, 95% CI = 1.28, 1.44, *p* < 0.001), liver (IRR = 1.14, 95% CI = 1.09, 1.19, *p* < 0.001), and stomach (IRR = 1.14, 95% CI = 1.09, 1.20, *p* < 0.001), cancers among Hispanic residents in PPAs.

At last, among non‐Hispanic White residents, we observed higher rates of cervical (IRR = 1.81, 95% CI = 1.64, 2.00, *p* < 0.001), colorectal (IRR = 1.17, 95% CI = 1.13, 1.20, *p* < 0.001), kidney (IRR = 1.13, 95% CI = 1.08, 1.19, *p* < 0.001), liver (IRR = 1.77, 95% CI = 1.68, 1.88, *p* < 0.001), lung (IRR = 1.46, 95% CI = 1.43, 1.50, *p* < 0.001), oropharyngeal (IRR = 1.28, 95% CI = 1.21, 1.34, *p <* 0.001), pancreatic (IRR = 1.06, 95% CI = 1.00, 1.12, *p* = 0.04), stomach (IRR = 1.26, 95% CI = 1.17, 1.36, *p* < 0.001), and uterine (IRR = 1.09, 95% CI = 1.03, 1.15, *p* = 0.003) cancers, and lower rates of female breast (IRR = 0.87, 95% CI = 0.84, 0.89, *p* < 0.001), melanoma (IRR = 0.68, 95% CI = 0.65, 0.71, *p <* 0.001), prostate (IRR = 0.88, 95% CI = 0.86, 0.91, *p <* 0.001), and thyroid (IRR = 0.75, 95% CI = 0.70, 0.80, *p <* 0.001) cancers in PPAs compared to non‐PPAs (Table 3).

### 3.3 Cancer Incidence Temporal Trends, 2006–2019

In Table 4, we show AAPCs among male and female residents living in PPAs and non‐PPAs, highlighting statistically significant incidence trends below. Melanoma increased over time among both males (AAPC = 1.1, 95% CI = 0.4, 1.8, *p* = 0.002) and females (AAPC = 1.3, 95% CI = 0.8, 1.8, *p* < 0.001) in non‐PPAs but did not significantly change among males and females in PPAs. Similarly, pancreatic cancer only increased in non‐PPAs among both males (AAPC = 0.6, 95% CI = 0.4, 0.8, *p* < 0.001) and females (AAPC = 0.5, 95% CI = 0.1, 1.0, *p* = 0.018). Non‐Hodgkin lymphoma incidence decreased among both males (AAPC = −0.6, 95% CI = −0.8, −0.3, *p* < 0.001) and females (AAPC = −0.5, 95% CI = −0.9, −0.2, *p* = 0.004) in non‐PPAs only. Specifically among females, cervical (AAPC = −0.9, 95% CI = −1.5, −0.3, *p* = 0.005) and oropharyngeal (AAPC = −0.8, 95% CI = −1.3, −0.3, *p* = 0.007) cancers decreased only in non‐PPAs, and kidney (AAPC = 0.9, 95% CI = 0.4, 1.3, *p* = 0.001) cancer increased only in non‐PPAs.

**TABLE 4:** Average annual percent change (AAPC) in age‐adjusted incidence rates by persistent poverty area (PPA) and sex, 2006–2019.

Sex/cancer type	PPA				Non‐PPA			
	AAPC	95% CI		*p*	AAPC	95% CI		*p*
*Male*								
Bladder	−3.1*	(−4.2, −2.1)	**▼**	< 0.001	−1.8*	(−2.2, −1.4)	**▼**	< 0.001
Breast	−1.7	(−4.2, 0.9)	—	0.179	−0.2	(−1.3, 0.9)	—	0.677
Colorectal	−1.4*	(−2.1, −0.8)	**▼**	< 0.001	−2.3*	(−3.6, −0.9)	**▼**	0.002
Kidney	0.7*	(0.1, 1.4)	▲	0.025	0.7*	(0.3, 1.1)	▲	0.005
Leukemia	−0.9	(−2.7, 0.9)	—	0.275	−0.4	(−1.1, 0.2)	—	0.2
Liver	1.5	(−0.1, 3.2)	—	0.073	0.4	(−0.2, 1.0)	—	0.227
Lung	−3.4*	(−4.0, −2.9)	**▼**	< 0.001	−3.4*	(−3.7, −3.2)	**▼**	< 0.001
Melanoma	0.5	(−0.6, 1.7)	—	0.32	1.1*	(0.4, 1.8)	▲	0.002
Non‐Hodgkin Lymphoma	1.0	(−1.2, 3.1)	—	0.378	−0.6*	(−0.8, −0.3)	**▼**	< 0.001
Oropharyngeal	−0.2	(−2.7, 2.3)	—	0.854	0.1	(−0.2, 0.5)	—	0.412
Pancreas	0.9	(−0.1, 2.0)	—	0.076	0.6*	(0.4, 0.8)	▲	< 0.001
Prostate	−3.4*	(−5.2, −1.6)	**▼**	< 0.001	−3.2*	(−5.5, −0.8)	**▼**	0.008
Stomach	−1.9*	(−2.8, −1.0)	**▼**	0.001	−1.3*	(−1.7, −0.9)	**▼**	< 0.001
Thyroid	3.6*	(2.3, 4.9)	▲	< 0.001	2.4*	(1.6, 3.2)	▲	< 0.001
*Female*								
Bladder	−1.8*	(−2.7, −0.9)	**▼**	0.001	−1.8*	(−2.2, −1.5)	**▼**	< 0.001
Breast	0.4	(0, 0.8)	—	0.054	0.1	(−0.2, 0.3)	—	0.555
Cervix	−2.0	(−5.4, 1.4)	—	0.248	−0.9*	(−1.5, −0.3)	**▼**	0.005
Colorectal	−1.4*	(−2.0, −0.7)	**▼**	< 0.001	−2.0*	(−2.4, −1.5)	**▼**	< 0.001
Kidney	1.2	(0, 2.4)	—	0.058	0.9*	(0.4, 1.3)	▲	0.001
Leukemia	−0.4	(−1.6, 0.8)	—	0.462	−0.3	(−1.0, 0.3)	—	0.291
Liver	2.1*	(0.6, 3.7)	▲	0.01	1.6*	(1.1, 2.2)	▲	< 0.001
Lung	−2.5*	(−3.2, −1.8)	**▼**	< 0.001	−2.3*	(−2.5, −2.0)	**▼**	< 0.001
Melanoma	0.9	(−1.0, 2.8)	—	0.315	1.3*	(0.8, 1.8)	▲	< 0.001
Non‐Hodgkin Lymphoma	0.4	(−0.7, 1.5)	—	0.442	−0.5*	(−0.9, −0.2)	**▼**	0.004
Oropharyngeal	−1.0	(−3.1, 1.1)	—	0.308	−0.8*	(−1.3, −0.3)	**▼**	0.007
Pancreas	−0.5	(−2.6, 1.5)	—	0.615	0.5*	(0.1, 1.0)	▲	0.018
Stomach	0.4	(−1.4, 2.3)	—	0.61	−0.2	(−0.7, 0.3)	—	0.435
Thyroid	4.0*	(2.3, 5.7)	▲	< 0.001	2.1*	(1.4, 2.9)	▲	< 0.001
Uterine	2.7*	(1.9, 3.5)	▲	< 0.001	1.7*	(1.5, 1.9)	▲	< 0.001

*Note:* **▼**: Statistically significant decrease. ▲: Statistically significant increase.

Abbreviations: —, change in incidence rate was not statistically significant; AAPC, average annual percent change. A positive AAPC means incidence rates increased; a negative AAPC means incidence rates decreased over the period; CI, confidence interval; Non‐PPA, non‐persistent poverty area; PPA, persistent poverty area.

* AAPC is significantly different from zero at *p* < 0.05.

In Table 5, we show AAPCs in PPAs and non‐PPAs by race/ethnicity. Among American Indian residents, increases were observed for most cancers in non‐PPAs, and prostate (AAPC = −2.8, 95% CI = −4.0, −1.5, *p* = 0.001) cancer significantly decreased only in non‐PPAs. Among Asian/Pacific Islander residents, cervical cancer decreased in both groups, with a much more pronounced decrease in PPAs (AAPC = −10.5, 95% CI = −18.5, −1.7, *p* = 0.021) versus non‐PPAs (AAPC = −1.3, 95% CI = ‐2.1, −0.5, *p* = 0.004). In non‐PPAs only, bladder (AAPC = −1.4, 95% CI = −2.1, −0.6, *p* = 0.002) and liver (AAPC = −2.8, 95% CI = −3.4, −2.1, *p* < 0.001) cancers decreased, whereas pancreatic (AAPC = 0.6, 95% CI = 0.3, 1.0, *p* = 0.001) and thyroid (AAPC = 2.0, 95% CI = 0.8, 3.1, *p* = 0.001) cancers increased.

**TABLE 5:** Average annual percent change (AAPC) in age‐adjusted incidence rates by persistent poverty area (PPA) and race/ethnicity, 2006–2019.

Race/ethnicity/cancer type	PPA				Non‐PPA			
	AAPC	95% CI		*p*	AAPC	95% CI		*p*
*American Indian*								
Bladder	−4.7	(−11.0, 2.1)	—	0.154	4.8*	(2.1, 7.5)	▲	0.002
Cervix	~	~	~	~	−0.6	(−7.2, 6.5)	—	0.849
Colorectal	3.5	(−1.3, 8.6)	—	0.141	1.2*	(0.3, 2.1)	▲	0.012
Female breast	0.7	(−2.6, 4.0)	—	0.654	3.8*	(1.4, 6.4)	▲	0.002
Kidney	~	~	~	~	3.9*	(1.6, 6.2)	▲	0.003
Leukemia	0.5	(−6.5, 8.0)	—	0.89	2.7	(−1.0, 6.4)	—	0.136
Liver	~	~	~	~	4.5*	(1.7, 7.2)	▲	0.004
Lung	−1.3	(−4.6, 2.1)	—	0.425	0.9	(−1.3, 3.1)	—	0.381
Melanoma	~	~	~	~	4.3*	(0.9, 7.8)	▲	0.016
Non‐Hodgkin lymphoma	2.4	(−2.9, 8.0)	—	0.352	1.5	(−0.9, 4.0)	—	0.189
Oropharyngeal	−6.3	(−12.7, 0.6)	—	0.068	2.0	(−0.6, 4.6)	—	0.123
Pancreas	~	~	~	~	6.1*	(1.6, 10.8)	▲	0.011
Prostate	−2.6	(−8.3, 3.3)	—	0.345	−2.8*	(−4.0, −1.5)	**▼**	0.001
Stomach	~	~	~	~	2.3	(−1.0, 5.6)	—	0.154
Thyroid	~	~	~	~	3.3	(−1.9, 8.7)	—	0.225
Uterine	4.8	(−1.4, 11.4)	—	0.121	4.8*	(1.5, 8.2)	▲	0.008
*Asian/Pacific Islander*								
Bladder	−0.5	(−3.7, 2.8)	—	0.735	−1.4*	(−2.1, −0.6)	**▼**	0.002
Cervix	−10.5*	(−18.5, −1.7)	**▼**	0.021	−1.3*	(−2.1, −0.5)	**▼**	0.004
Colorectal	−1.6*	(−2.7, −0.4)	**▼**	0.013	−2.8*	(−3.1, −2.5)	**▼**	< 0.001
Female breast	1.8*	(0.5, 3.0)	▲	0.008	1.3*	(0.9, 1.8)	▲	< 0.001
Kidney	1.7	(−0.9, 4.4)	—	0.182	0.6	(−0.2, 1.5)	—	0.128
Leukemia	−0.4	(−3.6, 2.9)	—	0.776	0.0	(−0.7, 0.7)	—	0.972
Liver	−2.1	(−4.1, 0.1)	—	0.055	−2.8*	(−3.4, −2.1)	**▼**	< 0.001
Lung	−2.1*	(−3.6, −0.6)	**▼**	0.01	−1.1*	(−1.4, −0.8)	**▼**	< 0.001
Melanoma	4.4	(−5.9, 15.9)	—	0.38	−0.9	(−2.4, 0.5)	—	0.188
Non‐Hodgkin lymphoma	0.6	(−1.6, 2.9)	—	0.546	0.1	(−0.4, 0.7)	—	0.571
Oropharyngeal	−0.3	(−3.0, 2.5)	—	0.82	−0.2	(−0.9, 0.5)	—	0.525
Pancreas	2.0	(0, 4.0)	—	0.051	0.6*	(0.3, 1.0)	▲	0.001
Prostate	−6.6*	(−8.5, −4.7)	**▼**	< 0.001	−3.7*	(−6.8, −0.6)	**▼**	0.02
Stomach	−3.4*	(−5.7, −1.0)	**▼**	0.009	−3.0*	(−3.8, −2.2)	**▼**	< 0.001
Thyroid	2.7	(−0.5, 6.1)	—	0.088	2.0*	(0.8, 3.1)	▲	0.001
Uterine	5.0*	(1.4, 8.7)	▲	0.01	2.4*	(1.9, 2.9)	▲	< 0.001
*Non‐Hispanic Black*								
Bladder	−1.5	(−3.6, 0.6)	—	0.141	−2.5*	(−4.3, −0.6)	**▼**	0.009
Cervix	−2.0	(−5.2, 1.2)	—	0.195	−0.4	(−2.1, 1.2)	—	0.586
Colorectal	−2.8*	(−4.1, −1.4)	**▼**	0.001	−3.4*	(−5.0, −1.7)	**▼**	< 0.001
Female breast	−0.4	(−1.3, 0.5)	—	0.354	−0.4	(−0.9, 0.2)	—	0.151
Kidney	0.6	(−1.4, 2.7)	—	0.534	0.5	(−0.8, 1.7)	—	0.425
Leukemia	−1.0	(−3.7, 1.7)	—	0.422	−0.4	(−1.6, 0.8)	—	0.482
Liver	−0.2	(−2.9, 2.6)	—	0.872	−0.6	(−1.5, 0.3)	—	0.208
Lung	−2.1*	(−3.0, −1.1)	**▼**	< 0.001	−3.2*	(−3.7, −2.7)	**▼**	< 0.001
Melanoma	~	~	~	~	−1.5	(−5.3, 2.5)	—	0.426
Non‐Hodgkin lymphoma	0.4	(−3.2, 4.1)	—	0.83	−0.4	(−1.4, 0.6)	—	0.386
Oropharyngeal	−3.2*	(−4.8, −1.7)	**▼**	0.001	−1.1*	(−2.1, −0.1)	**▼**	0.032
Pancreas	−0.5	(−2.1, 1.2)	—	0.54	−0.1	(−1.0, 0.8)	—	0.752
Prostate	−2.7*	(−4.8, −0.5)	**▼**	0.014	−3.7*	(−6.6, −0.7)	**▼**	0.017
Stomach	−2.5*	(−3.9, −1.2)	**▼**	0.001	−1.8*	(−2.8, −0.8)	**▼**	0.002
Thyroid	4.7*	(1.1, 8.4)	▲	0.015	0.5	(−1.5, 2.5)	—	0.643
Uterine	2.2*	(0.4, 4.1)	▲	0.019	2.7*	(1.8, 3.7)	▲	< 0.001
*Hispanic*								
Bladder	−1.5	(−3.8, 0.9)	—	0.193	−1.5*	(−2.1, −1.0)	**▼**	< 0.001
Cervix	−1.9	(−5.5, 1.8)	—	0.311	−2.0*	(−3.4, −0.6)	**▼**	0.005
Colorectal	0.4*	(0.0, 0.8)	▲	0.04	−1.4*	(−1.9, −0.9)	**▼**	< 0.001
Female breast	1.4*	(0.7, 2.1)	▲	0.001	0.8*	(0.5, 1.1)	▲	< 0.001
Kidney	1.9*	(1.0, 2.7)	▲	< 0.001	1.5*	(0.9, 2.2)	▲	< 0.001
Leukemia	0.3	(−0.9, 1.5)	—	0.574	−0.2	(−0.8, 0.3)	—	0.369
Liver	1.9	(−0.1, 4.0)	—	0.058	1.1*	(0.5, 1.7)	▲	0.001
Lung	−1.6*	(−2.7, −0.6)	**▼**	0.006	−2.3*	(−2.7, −1.9)	**▼**	< 0.001
Melanoma	2.0	(−0.4, 4.4)	—	0.097	1.0*	(0.1, 1.9)	▲	0.029
Non‐Hodgkin lymphoma	1.2*	(0.4, 2.1)	▲	0.01	−0.3	(−0.7, 0.1)	—	0.127
Oropharyngeal	−0.2	(−1.9, 1.6)	—	0.803	1.5*	(0.3, 2.7)	▲	0.012
Pancreas	0.3	(−1.5, 2.0)	—	0.755	0.7*	(0.2, 1.2)	▲	0.008
Prostate	−3.0*	(−5.0, −1.0)	**▼**	0.004	−3.6*	(−5.4, −1.8)	**▼**	< 0.001
Stomach	−0.2	(−1.2, 0.9)	—	0.689	−1.3*	(−1.8, −0.8)	**▼**	< 0.001
Thyroid	4.3*	(2.7, 6.0)	▲	< 0.001	3.0*	(2.1, 3.9)	▲	< 0.001
Uterine	4.4*	(3.5, 5.2)	▲	< 0.001	3.4*	(2.9, 3.8)	▲	< 0.001
*Non‐Hispanic White*								
Bladder	−2.8*	(−3.6, −2.0)	**▼**	< 0.001	−1.2*	(−1.6, −0.9)	**▼**	< 0.001
Cervix	1.4	(−0.7, 3.6)	—	0.17	−0.9*	(−1.5, −0.3)	**▼**	0.007
Colorectal	−2.0*	(−2.8, −1.3)	**▼**	< 0.001	−2.0*	(−2.8, −1.2)	**▼**	< 0.001
Female breast	−0.3	(−1.0, 0.4)	—	0.36	−0.1	(−0.3, 0.1)	—	0.275
Kidney	−0.4	(−1.4, 0.6)	—	0.375	0.4*	(0.1, 0.8)	▲	0.025
Leukemia	−1.7*	(−3.3, 0.0)	**▼**	0.045	−0.2	(−0.9, 0.5)	—	0.627
Liver	2.3*	(0.7, 3.9)	▲	0.009	1.3*	(0.4, 2.2)	▲	0.006
Lung	−3.1*	(−3.6, −2.5)	**▼**	< 0.001	−2.9*	(−3.1, −2.6)	**▼**	< 0.001
Melanoma	1.1*	(0.2, 2.0)	▲	0.016	1.5*	(0.3, 2.7)	▲	0.015
Non‐Hodgkin lymphoma	−2.2*	(−3.6, −0.8)	**▼**	0.005	−0.7*	(−1.0, −0.4)	**▼**	< 0.001
Oropharyngeal	−0.1	(−1.3, 1.1)	—	0.837	0.4*	(0.1, 0.6)	▲	0.017
Pancreas	0.7	(−0.3, 1.8)	—	0.155	0.8*	(0.2, 1.4)	▲	0.01
Prostate	−5.2*	(−6.5, −3.9)	**▼**	< 0.001	−3.4*	(−5.7, −0.9)	**▼**	0.007
Stomach	−0.8	(−3.7, 2.2)	—	0.556	−1.0*	(−1.5, −0.5)	**▼**	0.001
Thyroid	4.2	(−1.6, 10.5)	—	0.159	1.6*	(0.6, 2.6)	▲	0.002
Uterine	0.6	(−1.1, 2.2)	—	0.486	0.8*	(0.5, 1.1)	▲	< 0.001

*Note:* ▲: Statistically significant increase. **▼**: Statistically significant decrease.

Abbreviations: —, Change in rate was not statistically significant; ~, AAPC not calculated due to unstable incidence rates; AAPC, average annual percent change. A positive AAPC means incidence rates increased; a negative AAPC means incidence rates decreased over the per period; CI, confidence interval; non‐PPA: non‐persistent poverty area; PPA, persistent poverty area.

* AAPC is significantly different from zero at *p* < 0.05.

Among non‐Hispanic Black residents, bladder (AAPC = −2.5, 95% CI = −4.3, −0.6, *p* = 0.009) cancer decreased in non‐PPAs only and thyroid (AAPC = 4.7, 95% CI = 1.1, 8.4, *p* = 0.015) cancer increased in PPAs only (Table 5). Among Hispanic residents, bladder (AAPC = −1.5, 95% CI = −2.1, −1.0, *p* < 0.001), cervical (AAPC = −2.0, 95% CI = −3.4, −0.6, *p* = 0.005) and stomach (AAPC = −1.3, 95% CI = −1.8, −0.8, *p* < 0.001) cancers decreased only in non‐PPAs. Additionally, liver (AAPC = 1.1, 95% CI = 0.5, 1.7, *p* = 0.001), melanoma (AAPC = 1.0, 95% CI = 0.1, 1.9, *p* = 0.029), oropharyngeal (AAPC = 1.5, 95% CI = 0.3, 2.7, *p* = 0.012), and pancreatic (AAPC = 0.7, 95% CI = 0.2, 1.2, *p* = 0.008) cancers increased only in non‐PPAs, whereas non‐Hodgkin lymphoma (AAPC = 1.2, 95% CI = 0.4, 2.1, *p* = 0.01) increased only in PPAs. Colorectal cancer increased in PPAs (AAPC = 0.4, 95% CI = 0.0, 0.8, *p* = 0.04) and decreased in non‐PPAs (AAPC = −1.4, 95% CI = −1.9, −0.9, *p* < 0.001).

At last, among non‐Hispanic White residents, cervical (AAPC = −0.9, 95% CI = −1.5, −0.3, *p* = 0.007) and stomach (AAPC = −1.0, 95% CI = −1.5, −0.5, *p* = 0.001) cancers significantly decreased over time in non‐PPAs only, and leukemia (AAPC = −1.7, 95% CI = −3.3, 0.0, *p* = 0.045) decreased in PPAs only (Table 5). Interestingly, kidney (AAPC = 0.4, 95% CI = 0.1, 0.8, *p* = 0.025), oropharyngeal (AAPC = 0.4, 95% CI = 0.1, 0.6, *p* = 0.017), pancreatic (AAPC = 0.8, 95% CI = 0.2, 1.4, *p* = 0.01), thyroid (AAPC = 1.6, 95% CI = 0.6, 2.6, *p* = 0.002), and uterine (AAPC = 0.8, 95% CI = 0.5, 1.1, *p* < 0.001) cancers significantly increased in non‐PPAs.

## 4 Discussion

In this population‐based study, we observed higher incidence rates for several cancers in PPAs including cervical, liver, lung, and stomach cancers, and lower incidence rates for breast, melanoma, prostate, and thyroid cancers compared to non‐PPAs. Consistent with previous studies in California and in the United States, we found that PPAs had lower percentages of non‐Hispanic White patients and higher percentages of non‐Hispanic Black and Hispanic patients [8, 31]. Additionally, most PPA residents who were diagnosed with cancer were either uninsured or had public insurance. These observations highlight the potential for racial/ethnic and socioeconomic barriers to screening for cancer precursors and prevention services, which may be contributing to the increased incidence of some cancer types in PPAs [15].

Our findings of significantly higher liver and cervical cancer incidence rates among all racial/ethnic groups studied, and higher stomach cancer incidence rates among Asian/Pacific Islander, non‐Hispanic Black, Hispanic, and non‐Hispanic White individuals in PPAs compared to non‐PPAs emphasize the need for additional research to identify etiological factors and any systemic barriers to preventive services in PPAs [16]. Of the cancers we examined, cervical, liver, and stomach cancers are the three cancers most strongly associated with infections, with established links between human papillomavirus (HPV) and cervical cancer, hepatitis C and B viruses and liver cancer, and *Helicobacter pylori* (*H. pylori*) infection and stomach cancer [32, 33]. The higher incidence of infection‐related cancers in PPAs may indicate barriers to screening for HPV, *H. pylori*, hepatitis C and B infections, as the identification and eradication of these infections in precancerous stages may prevent their progression into malignant tumors [32, 34]. Liver cancer is also linked to alcoholic and metabolic dysfunction‐associated steatotic liver disease (MASLD). MASLD is associated with obesity and diabetes and is becoming the most common cause of chronic liver disease in the US [35, 36, 37, 38]. The higher incidence of obesity‐related cancers in PPAs may also be related to the higher rates of food insecurity and difficulty accessing nutritious foods in PPA food deserts [39]. The quality and affordability of healthy food options in PPAs are important topics to investigate to better understand the complex relationship between diet and cancer risk among residents in PPAs [40]. Additionally, cervical, liver, lung, and stomach cancers are all tobacco‐related, and our findings may relate to higher tobacco use in PPAs [41].

We also observed higher rates of additional tobacco‐related cancers including colorectal cancers, among American Indian, non‐Hispanic Black, non‐Hispanic White, and Asian/Pacific Islander individuals in PPAs. These findings may indicate ongoing disparities related to tobacco use, as previous studies have identified higher rates of tobacco use among US adults below the poverty line compared to those at or above the poverty line across diverse racial and ethnic groups [41]. Additionally, low income and living in higher poverty neighborhoods are risk factors for tobacco use [42, 43]. Educational attainment is also lower in poverty areas, and lower education has been linked with higher prevalence of risky behaviors related to cancer including tobacco use [7, 41].

Although previous studies have described higher rates of kidney cancer among American Indian populations compared to other racial/ethnic groups, our study shows that in California, American Indian residents in PPAs have even higher rates of kidney cancer compared to those residing in non‐PPAs [44]. Several modifiable risk factors for kidney cancer including obesity and tobacco use, as well as a diagnosis of type 2 diabetes or hypertension, may be contributing to the higher rates of kidney cancer for American Indian residents in PPAs [45, 46].

Our findings of lower rates of most cancers we studied among Asian/Pacific Islander and Hispanic residents in PPAs may relate, in part, to the healthy immigrant effect, as 41% of Hispanic and 58% of Asian/Pacific Islander patients in PPAs were foreign‐born compared to 31% and 48% in non‐PPAs. The healthy immigrant effect [47] suggests that people who migrate have better overall health compared to people from their native and adopted countries. Additionally, acculturation has been linked with increased high‐risk behaviors associated with cancer including alcohol use, smoking, and a diet high in fatty or processed foods [48, 49]. As such, the lower incidence of several cancers in race/ethnic groups with a high percentage of foreign‐born patients may suggest protective sociocultural behaviors associated with their foreign‐born status such as living in enclaves and having access to healthy cultural foods, greater social support, and lower engagement in high‐risk behaviors [49]. However, further research considering nativity, genetics, diet, tobacco, alcohol use, and environmental factors is needed to further understand specific variables underlying these findings.

The increasing rates of non‐Hodgkin lymphoma we observed among Hispanic patients in PPAs may suggest environmental disparities, as previous research has shown that environmental risk factors for non‐Hodgkin lymphoma, including exposure to pesticides, may disproportionally affect Hispanic and African American individuals living in urban areas [50]. On the other hand, the decreasing rates of cervical cancer among Asian/Pacific Islander individuals in PPAs and non‐PPAs alike may point to successful public health efforts, such as the availability of medical interpretation services and culturally tailored resources, and can serve as models for reducing cervical cancer incidence among the other racial/ethnic minority groups [51, 52, 53].

The lower incidence of several cancers, including thyroid, female breast, prostate, and melanoma in PPAs is consistent with previous research observing a higher incidence of female breast, prostate, and melanoma in those residing in higher socioeconomic status neighborhoods [1, 54, 55]. The higher incidence of breast cancer among women in non‐PPAs may be related to health behavior differences compared to women in PPAs. For example, hormone replacement therapy (HRT) to manage menopause symptoms is more commonly used among affluent and non‐Hispanic White women, and previous studies have shown a link between HRT use and breast cancer [56, 57]. This finding may also be related to a previously established link between older age at first birth and breast cancer, as women with higher socioeconomic status have a later age at first birth compared to women with lower socioeconomic status [54, 58]. Our observation of higher prostate cancer incidence among men in non‐PPAs is consistent with previous studies showing a positive relationship between higher socioeconomic status and prostate cancer diagnosis, possibly due to better access to health services and higher prostate screening rates among affluent men [55, 59, 60]. Additionally, the significantly lower rates of melanoma among male and female residents in PPAs, and the significantly increasing rates over time of melanoma observed among male and female residents in non‐PPAs, are consistent with established evidence of an association between higher income in the United States and higher risk for melanoma [61]. This association has been attributed to risk factors among individuals with higher income, including exposure to ultraviolet radiation through recreational tanning and use of tanning beds [62]. As such, the lower incidence of melanoma among PPAs may be primarily associated with race/ethnicity and income, as non‐Hispanic White individuals are at higher risk for developing melanoma and most non‐Hispanic White patients in our study resided in non‐PPAs [63].

Cancer incidence trends by race/ethnicity and sex are indicative that public health efforts to reduce cancer incidence may be disproportionately benefiting patients in non‐PPAs, as non‐Hodgkin lymphoma decreased only among males and females in non‐PPAs, and oropharyngeal and cervical cancer decreased only among females in non‐PPAs. The incidence of several cancers decreasing only among residents of non‐PPAs was also observed among specific racial/ethnic groups, including colorectal cancer among Hispanic residents, cervical and stomach cancer among Hispanic and non‐Hispanic White residents, bladder cancer among non‐Hispanic Black, Asian/Pacific Islander, and Hispanic residents, liver cancer among Asian/Pacific Islander residents, and prostate cancer among American Indian residents. These trends suggest that public health interventions, including tobacco cessation and vaccination programs, may be effective, but patients in PPAs may face additional barriers to receiving these interventions. Recently identified barriers to cancer prevention and control efforts for patients in PPAs include lack of transportation and available health providers, patient uncertainty of research benefits, and patient health literacy [18]. Strategies to reduce these burdens include integrating cancer education with community health events, reducing transportation and cost barriers, and standardizing patient navigation in research and clinical settings [18]. Further, the increasing rates of several cancers in non‐PPAs, including pancreatic cancer among males and females, and kidney cancer among females, suggest the need for additional research into the underlying etiologic factors that may explain these differences.

Our study is subject to some limitations. Previous concerns have been raised about the possible misclassification of the poverty status of census tracts with large off‐campus college student populations, as they are included in area poverty measures and are a transient resident population [64]. However, including census tracts with large off‐campus college student populations may be an important part of understanding the relationship between poverty and health disparities, as recent reports describe overwhelming evidence for college student poverty in California [65, 66, 67]. In 2023, the California Student Aid Commission reported that 66% of college students across California's higher education systems, including University of California, California State University, California Community College, private non‐profit, and private for‐profit schools, were food insecure and 53% were housing insecure [68]. Additionally, among all students experiencing housing insecurity, students in off‐campus housing had the highest expenses [68]. These concerns remain even for dependent college students and students receiving financial support from family, guardians, or relatives, as 41% and 27% were housing insecure [68]. Further, a report examining the impact of excluding off‐campus college students on poverty rates in United States counties found that this exclusion only modestly impacted poverty rates at finer levels of geography, including county and city levels [64], unlike our study that presents findings at the state level. Reporting only the AAPC in cancer incidence rates is another limitation, as it may not accurately reflect any underlying changes in trends over time. However, the AAPC is recommended in small demographics to mitigate unstable incidence rates, as was the case in our analyses stratified by race/ethnicity and cancer type [28, 29, 69]. For example, due to small numbers in the American Indian population in PPAs, we were unable to calculate temporal trends for numerous cancers, including cervical, kidney, liver, melanoma, pancreatic, stomach, and thyroid cancers. Therefore, our understanding of trends for these cancers by race/ethnicity is incomplete. Future studies with larger populations should also consider annual percent change for each segment of the study period where significant trend changes occur.

Despite these limitations, this population‐based study is the first to report incidence rates and trends for common cancers by PPAs in California by race/ethnicity and sex. Our findings reveal cancer disparities in PPAs that may be used to develop and target interventions to reduce the impact of poverty on cancer. Policy changes, such as increasing the minimum wage and strengthening public support programs like CalWORKs and CalFresh, can decrease the health disparities experienced by Californians in PPAs by helping pay for housing, increasing access to fresh fruits and vegetables, and providing nutrition education [70, 71, 72]. Additionally, our findings provide important insight for national efforts to combat health disparities in PPAs. For example, the Persistent Poverty Initiative, coordinated by the NCI, was launched in 2023 as the first major program to address structural and institutional factors of persistent poverty and cancer disparities [73]. Our study findings specific to PPAs in California can further guide the implementation of this initiative in diverse racial/ethnic populations in the United States.

## 5 Conclusion

In summary, we identified disparities in cancer incidence for several cancer types in PPAs, including higher infection and tobacco‐related cancers. Culturally sensitive public health efforts to increase access to preventive measures, including tobacco‐cessation programs, HPV and Hepatitis B vaccinations, and early detection and treatment of hepatitis C and *H. pylori*, are needed in PPAs to decrease the incidence of infection‐associated cancers [32]. Research‐based public health initiatives for residents in economically disadvantaged areas are needed to better serve California's vulnerable populations.

## Author Contributions

**Ani S. Movsisyan Vernon:** conceptualization (lead), data curation (lead), formal analysis (lead), investigation (lead), methodology (lead), project administration (equal), validation (lead), visualization (lead), writing – original draft (lead), writing – review and editing (lead). **Frances B. Maguire:** conceptualization (equal), data curation (equal), formal analysis (equal), investigation (equal), methodology (equal), validation (equal), writing – original draft (equal), writing – review and editing (equal). **Ayman T. Ullah:** writing – original draft (supporting), writing – review and editing (supporting). **Brenda M. Hofer:** conceptualization (supporting), data curation (supporting), methodology (supporting), writing – review and editing (supporting). **Arti Parikh‐Patel:** conceptualization (supporting), writing – review and editing (supporting). **Ted Wun:** writing – review and editing (supporting). **Shehnaz K. Hussain:** conceptualization (equal), project administration (lead), supervision (lead), writing – review and editing (supporting). **Theresa H. M. Keegan:** conceptualization (equal), project administration (lead), supervision (lead), writing – review and editing (equal).

## Disclosure

Précis: Our study identified disparities in cancer incidence for several cancer types in persistent poverty areas (PPAs), including higher infection and tobacco‐related cancers. Culturally sensitive public health efforts to increase access to preventive measures are needed in PPAs.

## Ethics Statement

All analyses were overseen by the Institutional Review Board of the University of California, Davis. All methods were carried out in accordance with relevant guidelines and regulations.

## Conflicts of Interest

The authors declare no conflicts of interest.

## Data Availability

The data that supports the findings of this study are available from the California Department of Public Health and the California Cancer Registry. Access is granted through an application process by the management or data custodians (https://www.cdph.ca.gov/Programs/CHSI/Pages/Data‐Applications.aspx) and (https://www.ccrcal.org/retrieve‐data/).

## References

[1] D. L. Oh , K. Schumacher , J. Yang , et al., “Disparities in Cancer Incidence by Rurality in California,” JNCI Journal of the National Cancer Institute 115 (2023): 385–393. 36622036 10.1093/jnci/djac238 PMC10086626

[2] S. Bohn , C. Danielson , S. Kimberlin , P. Malagon , and C. Wimer , “Poverty in California,” (2023), https://www.ppic.org/publication/poverty‐in‐california.

[3] USDA ERS , “Poverty Area Measures,” https://www.ers.usda.gov/data‐products/poverty‐area‐measures/poverty‐area‐measures/.

[4] National Cancer Institute. Persistent Poverty and Cancer , “Increasing Health Equity Across the Cancer Continuum 1,” (2022), https://www.cancer.gov/research/annual‐plan/scientific‐topics/increasing‐health‐equity.

[5] C. L. Beale , “The Ethnic Dimension of Persistent Poverty in Rural and Small‐Town Areas,” Rural Minority Trends and Progress 1–7 (1996).

[6] J. L. Moss , C. N. Pinto , S. Srinivasan , K. A. Cronin , and R. T. Croyle , “Enduring Cancer Disparities by Persistent Poverty, Rurality, and Race,” JNCI Journal of the National Cancer Institute 114 (2022): 829–835. 35238347 10.1093/jnci/djac038 PMC9194626

[7] M. Doose , A. E. Kennedy , S. D. Williams , and S. Srinivasan , “The Context of Poverty and Cancer: Denying Human Potential,” Cancer Epidemiology, Biomarkers & Prevention 33 (2024): 1402–1404. 10.1158/1055-9965.EPI-24-0953 39482971

[8] J. L. Moss , C. N. Pinto , S. Srinivasan , K. A. Cronin , and R. T. Croyle , “Persistent Poverty and Cancer Mortality Rates: An Analysis of County‐Level Poverty Designations,” Cancer Epidemiology, Biomarkers & Prevention 29 (2020): 1949–1954. 10.1158/1055-9965.EPI-20-0007 PMC7534551 32998949

[9] S. Lin Gomez , S. Shariff‐Marco , and I. Cheng , “The Unrelenting Impact of Poverty on Cancer: Structural Inequities Call for Research and Solutions on Structural Determinants,” Journal of the National Cancer Institute 114 (2022): 783–784. 35172011 10.1093/jnci/djac040 PMC9194620

[10] J. R. Biden , “Executive Order on Advancing Racial Equity and Support for Underserved Communities Through the Federal Government,” The White House 1, (2021), https://www.whitehouse.gov/briefing‐room/presidential‐actions/2021/01/20/executive‐order‐advancing‐racial‐equity‐and‐support‐for‐underserved‐communities‐through‐the‐federal‐government/.

[11] UCSF Health Atlas , “University of California, San Francisco School of Medicine Dean's Office of Population Health and Health Equity,” (2022), https://healthatlas.ucsf.edu/?active=persistent_poverty&active&geography=tracts&tracts=&zoom=5.386384825310189&lng=‐119.9143703628364&lat=37.48219962671807.

[12] C. Danielson , P. Malagon , and S. Bohn , “Poverty in California,” Public Policy Institute of California 2, (2022), https://www.ppic.org/wp‐content/uploads/JTF_PovertyJTF.pdf.

[13] S. Gehlert , D. Hudson , and T. Sacks , “A Critical Theoretical Approach to Cancer Disparities: Breast Cancer and the Social Determinants of Health,” Frontiers in Public Health 9 (2021): 623. 10.3389/fpubh.2021.674736 PMC8175790 34095075

[14] D. Schillinger , “The Intersections Between Social Determinants of Health, Health Literacy, and Health Disparities,” Studies in Health Technology and Informatics 269 (2020): 22–41. 32593981 10.3233/SHTI200020 PMC7710382

[15] J. M. Hall , S. M. Szurek , H. Cho , et al., “Cancer Disparities Related to Poverty and Rurality for 22 Top Cancers in Florida,” Preventive Medical Reports 29 (2022): 101922. 10.1016/j.pmedr.2022.101922 PMC9344025 35928594

[16] F. P. Boscoe , C. J. Johnson , R. L. Sherman , D. G. Stinchcomb , G. Lin , and K. A. Henry , “The Relationship Between Area Poverty Rate and Site‐Specific Cancer Incidence in the United States,” Cancer 120 (2014): 2191–2199. 24866103 10.1002/cncr.28632 PMC4232004

[17] M. V. Papageorge , A. P. Woods , S. W. L. de Geus , et al., “The Persistence of Poverty and Its Impact on Cancer Diagnosis, Treatment and Survival,” Annals of Surgery 277 (2023): 995–1001. 35796386 10.1097/SLA.0000000000005455

[18] E. Hallgren , K. H. K. Yeary , P. DelNero , et al., “Barriers, Facilitators, and Priority Needs Related to Cancer Prevention, Control, and Research in Rural, Persistent Poverty Areas,” Cancer Causes & Control 34 (2023): 1145–1155. 37526781 10.1007/s10552-023-01756-1 PMC10547626

[19] “About California Cancer Registry—California Cancer Registry,” https://www.ccrcal.org/learn‐about‐ccr/about‐cancer‐registries/.

[20] California Cancer Registry & Chronic Disease Surveillance and Research Branch , “Policies and Procedures for Access to and Disclosure of Confidential Data From the California Cancer Registry January 2014,” 2 (2014).

[21] J. Hofferkamp , “Standards for Cancer Registries Volume III,” (2008).

[22] “Site Recode ICD‐O‐3/WHO 2008—SEER Data Reporting Tools,” https://seer.cancer.gov/siterecode/icdo3_dwhoheme/index.html.

[23] National Cancer Institute , “Poverty | Division of Cancer Control and Population Sciences (DCCPS),” (2024), https://cancercontrol.cancer.gov/hdhe/research‐emphasis/poverty.

[24] California , “Populations—Total U.S. (2006–2020), Census Tract Estimates by Race/Origin Controlling to Vintage 2020 <2010 Tract Geographies>, National Cancer Institute, DCCPS, Surveillance Research Program, Released January 2023. Source: Woods & Poole Economics, Inc.,” (2022), https://www.census.gov/geographies/reference‐files/2010/geo/state‐local‐geo‐guides‐2010/california.html.

[25] A. S. Movsisyan , “The Burden of Cancer in Persistent Poverty Areas of California,” (2023).

[26] “SEER*Stat Software,” https://seer.cancer.gov/seerstat/.

[27] “Joinpoint—Joinpoint Help System,” https://surveillance.cancer.gov/help/joinpoint/front‐page.

[28] “Average Annual Percent Change (AAPC) and Confidence Interval—Joinpoint Help System,” https://surveillance.cancer.gov/help/joinpoint/setting‐parameters/method‐and‐parameters‐tab/apc‐aapc‐tau‐confidence‐intervals/average‐annual‐percent‐change‐aapc/#Relative_Advantages_and_disadvantages_AAPC_vs_APCbookmark.

[29] “AAPC Definition—Joinpoint Help System,” https://surveillance.cancer.gov/help/joinpoint/tech‐help/frequently‐asked‐questions/aapc‐definition.

[30] A. B. Mariotto , E. J. Feuer , N. Howlader , H. S. Chen , S. Negoita , and K. A. Cronin , “Interpreting Cancer Incidence Trends: Challenges due to the COVID‐19 Pandemic,” JNCI Journal of the National Cancer Institute 115 (2023): 1109–1111. 37220901 10.1093/jnci/djad086 PMC10483261

[31] C. Wimer , M. Mattingly , M. Levin , C. Danielson , and S. Bohn , “A Portrait of Poverty Within California Counties and Demographic Groups,” The California Poverty Measure (2011).

[32] K. D. Volesky‐Avellaneda , S. Morais , S. D. Walter , et al., “Cancers Attributable to Infections in the US in 2017 A Meta‐Analysis Supplemental Content,” JAMA Oncology 9, no. Suppl (2023): 1678–1687. 37856141 10.1001/jamaoncol.2023.4273 PMC10587828

[33] A. Jemal , L. Torre , W. Street , and F. Bray , “The Cancer Atlas,” (2019).

[34] S. De Flora and P. Bonanni , “The Prevention of Infection‐Associated Cancers,” Carcinogenesis 32 (2011): 787–795. 21436188 10.1093/carcin/bgr054 PMC3314281

[35] J. L. Petrick and K. A. Mcglynn , “The Changing Epidemiology of Primary Liver Cancer,” Cancer Epidemiology 6 (2019): 104–111. 10.1007/s40471-019-00188-3 PMC6599615 31259140

[36] Z. M. Younossi , M. Stepanova , J. Ong , et al., “Nonalcoholic Steatohepatitis Is the Most Rapidly Increasing Indication for Liver Transplantation in the United States,” Clinical Gastroenterology and Hepatology 19 (2021): 580–589.e5. 32531342 10.1016/j.cgh.2020.05.064

[37] Z. M. Younossi , M. Stepanova , Y. Younossi , et al., “Epidemiology of Chronic Liver Diseases in the USA in the Past Three Decades,” Gut 69 (2020): 564–568. 31366455 10.1136/gutjnl-2019-318813

[38] M. E. Rinella and S. Sookoian , “From NAFLD to MASLD: Updated Naming and Diagnosis Criteria for Fatty Liver Disease,” Journal of Lipid Research 65 (2023): 100485. 38103785 10.1016/j.jlr.2023.100485 PMC10824973

[39] A. Odoms‐Young , A. G. M. Brown , T. Agurs‐Collins , and K. Glanz , “Food Insecurity, Neighborhood Food Environment, and Health Disparities: State of the Science, Research Gaps and Opportunities,” American Journal of Clinical Nutrition 119 (2024): 850–861. 38160801 10.1016/j.ajcnut.2023.12.019 PMC10972712

[40] R. C. Woodruff , R. Haardörfer , I. G. Raskind , A. Hermstad , and M. C. Kegler , “Comparing Food Desert Residents With Non‐Food Desert Residents on Grocery Shopping Behaviours, Diet and BMI: Results From a Propensity Score Analysis,” Public Health Nutrition 23 (2020): 806–811. 31957629 10.1017/S136898001900363X PMC8924878

[41] B. E. Garrett , B. N. Martell , R. S. Caraballo , and B. A. King , “Socioeconomic Differences in Cigarette Smoking Among Sociodemographic Groups,” Preventing Chronic Disease 16 (2019): E74. 31198164 10.5888/pcd16.180553 PMC6583815

[42] C. Cambron , R. Kosterman , and J. D. Hawkins , “Neighborhood Poverty Increases Risk for Cigarette Smoking From Age 30–39,” Annals of Behavioral Medicine 53 (2019): 858–864. 30395158 10.1093/abm/kay089 PMC6696901

[43] F. B. Maguire , A. S. Movsisyan , T. D. Vuong , et al., “Burden of Tobacco‐Related Cancers in California, 1988–2017,” (2020).

[44] P. S. Pinheiro , K. E. Callahan , S. L. Gomez , et al., “High Cancer Mortality for US‐Born Latinos: Evidence From California and Texas,” BMC Cancer 17 (2017): 1–13. 28693448 10.1186/s12885-017-3469-0 PMC5504850

[45] V. Gonzalez , M. Suflita , A. Janitz , et al., “Kidney Cancer Incidence and Mortality Disparities Involving American Indians/Alaska Natives: An Analysis of the Oklahoma Central Cancer Registry (OCCR),” Journal of Cancer Epidemiology 2022 (2022): 1–10, 10.1155/2022/2689386. PMC9234047 35769890

[46] T. B. Kratzer , A. Jemal , K. D. Miller , et al., “Cancer Statistics for American Indian and Alaska Native Individuals, 2022: Including Increasing Disparities in Early Onset Colorectal Cancer,” CA: A Cancer Journal for Clinicians 73 (2023): 120–146. 36346402 10.3322/caac.21757

[47] E. C. Dee and S. L. Gomez , “Cancer Among Immigrants: Diverse Histories, Diverse Disparities, Diverse Opportunities to Promote Equity,” Cancer Epidemiology, Biomarkers & Prevention 31 (2022): 1251–1253. 10.1158/1055-9965.EPI-22-0337 35775230

[48] M. C. Stern , L. Fejerman , R. das , et al., “Variability in Cancer Risk and Outcomes Within US Latinos by National Origin and Genetic Ancestry,” Current Epidemiology Reports 3 (2016): 181–190. 27547694 10.1007/s40471-016-0083-7 PMC4978756

[49] T. H. M. Keegan , T. Quach , S. Shema , S. L. Glaser , and S. L. Gomez , “The Influence of Nativity and Neighborhoods on Breast Cancer Stage at Diagnosis and Survival Among California Hispanic Women,” BMC Cancer 10 (2010): 1–11. 21050464 10.1186/1471-2407-10-603 PMC2988754

[50] D. Blansky , I. Mantzaris , T. Rohan , H. Dean , and H. Iii , “The Influence of Rurality, Race and Ethnicity on Non‐Hodgkin Lymphoma Incidence,” Clinical Lymphoma, Myeloma & Leukemia 20 (2020): 668–676. 10.1016/j.clml.2020.05.010 PMC7976043 32605898

[51] J. Dang , J. Lee , J. H. Tran , et al., “The Role of Medical Interpretation on Breast and Cervical Cancer Screening Among Asian American and Pacific Islander Women,” Journal of Cancer Education 25 (2010): 253–262. 20352398 10.1007/s13187-010-0074-1 PMC2878591

[52] California Department of Health Care Services , “Health Disparities in the Medi‐Cal Population,” (2015).

[53] C. A. Thompson , S. L. Gomez , A. Chan , et al., “Patient and Provider Characteristics Associated With Colorectal, Breast, and Cervical Cancer Screening Among Asian Americans,” Cancer Epidemiology, Biomarkers & Prevention 23 (2014): 2208–2217. 10.1158/1055-9965.EPI-14-0487 PMC4221799 25368396

[54] S. A. Robert , I. Strombom , A. Trentham‐Dietz , et al., “Socioeconomic Risk Factors for Breast Cancer: Distinguishing Individual‐ and Community‐Level Effects,” Epidemiology 15 (2004): 442–450. 15232405 10.1097/01.ede.0000129512.61698.03

[55] A. Rundle , K. M. Neckerman , D. Sheehan , et al., “A Prospective Study of Socioeconomic Status, Prostate Cancer Screening and Incidence Among Men at High Risk for Prostate Cancer,” Cancer Causes & Control 24 (2013): 297–303. 23224323 10.1007/s10552-012-0108-6 PMC3557724

[56] C. R. Cameron , S. Cohen , K. Sewell , and M. Lee , “The Art of Hormone Replacement Therapy (HRT) in Menopause Management,” Journal of Pharmacy Practice 37 (2024): 736–740. 37002679 10.1177/08971900231167925

[57] T. K. Iyer and J. A. E. Manson , “Recent Trends in Menopausal Hormone Therapy Use in the US: Insights, Disparities, and Implications for Practice,” JAMA Health Forum 5 (2024): e243135. 39331374 10.1001/jamahealthforum.2024.3135

[58] T. Van Roode , K. Sharples , N. Dickson , and C. Paul , “Life‐Course Relationship Between Socioeconomic Circumstances and Timing of First Birth in a Birth Cohort,” PLoS One 12 (2017): 1–16. 10.1371/journal.pone.0170170 PMC5234805 28085935

[59] S. S. Coughlin , “A Review of Social Determinants of Prostate Cancer Risk, Stage, and Survival,” Prostate International 8 (2019): 49. 32647640 10.1016/j.prnil.2019.08.001 PMC7335972

[60] J. L. Jahn , E. L. Giovannucci , and M. J. Stampfer , “The High Prevalence of Undiagnosed Prostate Cancer at Autopsy: Implications for Epidemiology and Treatment of Prostate Cancer in the Prostate‐Specific Antigen‐Era,” International Journal of Cancer 137 (2015): 2795–2802. 25557753 10.1002/ijc.29408 PMC4485977

[61] A. J. Jiang , P. V. Rambhatla , and M. J. Eide , “Socioeconomic and Lifestyle Factors and Melanoma: A Systematic Review,” British Journal of Dermatology 172 (2015): 885–915. 25354495 10.1111/bjd.13500

[62] J. E. McWhirter and L. Hoffman‐Goetz , “Coverage of Skin Cancer and Recreational Tanning in North American Magazines Before and After the Landmark 2006 International Agency for Research on Cancer Report,” BMC Public Health 15 (2015): 1–11. 25884779 10.1186/s12889-015-1511-1 PMC4342877

[63] A. P. Thrift and F. J. Gudenkauf , “Melanoma Incidence Among Non‐Hispanic Whites in All 50 US States From 2001 Through 2015,” JNCI Journal of the National Cancer Institute 112 (2020): 533–539. 31346623 10.1093/jnci/djz153 PMC7225671

[64] A. Bishaw , Examining the Effect of Off‐Campus College Students on Poverty Rates (U.S. Census Bureau, 2013).

[65] D. Burns , D. Espinoza , N. Ondrasek , and M. Yang , “California's Students Experiencing Homelessness: Conditions, Outcomes, and Policy Considerations,” (2021).

[66] “Real College California: Basic Needs Among California Community College Students,” (2023).

[67] Assembly Budget Committee , “California Student Housing: Solutions for Improving Capacity and Affordability,” (2021).

[68] California Student Aid Commission , “Food and Housing Survey: Understanding Students' Basic Needs,” (2023).

[69] L. X. Clegg , B. F. Hankey , R. Tiwari , E. J. Feuer , and B. K. Edwards , “Estimating Average Annual per Cent Change in Trend Analysis,” Statistics in Medicine 28 (2009): 3670–3682. 19856324 10.1002/sim.3733 PMC2843083

[70] California Budget & Policy Center , “Strategies to Reduce Poverty in California,” (2015).

[71] CalFresh , https://www.cdss.ca.gov/calfresh.

[72] CalWORKS , https://www.cdss.ca.gov/calworks.

[73] “RFA‐CA‐22‐015: Cancer Control Research in Persistent Poverty Areas (U54 Clinical Trial Optional),” https://grants.nih.gov/grants/guide/rfa‐files/RFA‐CA‐22‐015.html.

